# Surgical Triumph Over Metatarsal Osteosarcoma: A Rare Case Report Revealing Diagnostic Challenges and Successful Management

**DOI:** 10.7759/cureus.40987

**Published:** 2023-06-26

**Authors:** Sandeep Ghosh, Amar Jain, Deepak Bhojwani, Soumya Singh

**Affiliations:** 1 Surgical Oncology, Chirayu Medical College & Hospital, Bhopal, IND; 2 Anaesthesiology, Chirayu Medical College & Hospital, Bhopal, IND

**Keywords:** non-vascularized fibula graft, foot tumours, metatarsal tumour, chondroblastic osteosarcoma, bone and soft-tissue sarcoma

## Abstract

Osteosarcoma of the foot is exceedingly uncommon, and as a result, very little is known about patient and tumor characteristics. In addition, the prognosis may be grim due to delayed presentation and misdiagnosis. A delayed diagnosis of osteosarcoma, regardless of location, may not only reduce long-term survival but also modify the treatment plan, resulting in less favorable functional and cosmetic outcomes. Here we report the diagnostic and therapeutic challenges associated with chondroblastic osteosarcoma involving the metatarsal bone of the foot in a 47-year-old woman treated with wide local excision with right second metatarsectomy and non-vascularized fibular graft reconstruction along with adjuvant chemotherapy.

## Introduction

Foot and small bone osteosarcomas are a rare and distinct clinical entity. The incidence of foot osteosarcomas is estimated to range between 0.2% and 2% [1]. Approximately 32 cases of metatarsal osteosarcomas have been published in the medical literature from 1940 to 2018 [2]. Notably, a Mayo Clinic systematic review article identified the calcaneum as the most commonly affected bone in the foot [3]. The purpose of this case report is to describe the clinical presentation, diagnostic evaluation, surgical intervention, and ensuing outcome of a 47-year-old lady diagnosed with chondroblastic osteosarcoma of the second metatarsal. Through this report, we hope to contribute valuable knowledge to the comprehension and treatment of this uncommon presentation of foot osteosarcoma.

## Case presentation

**Figure 1:**
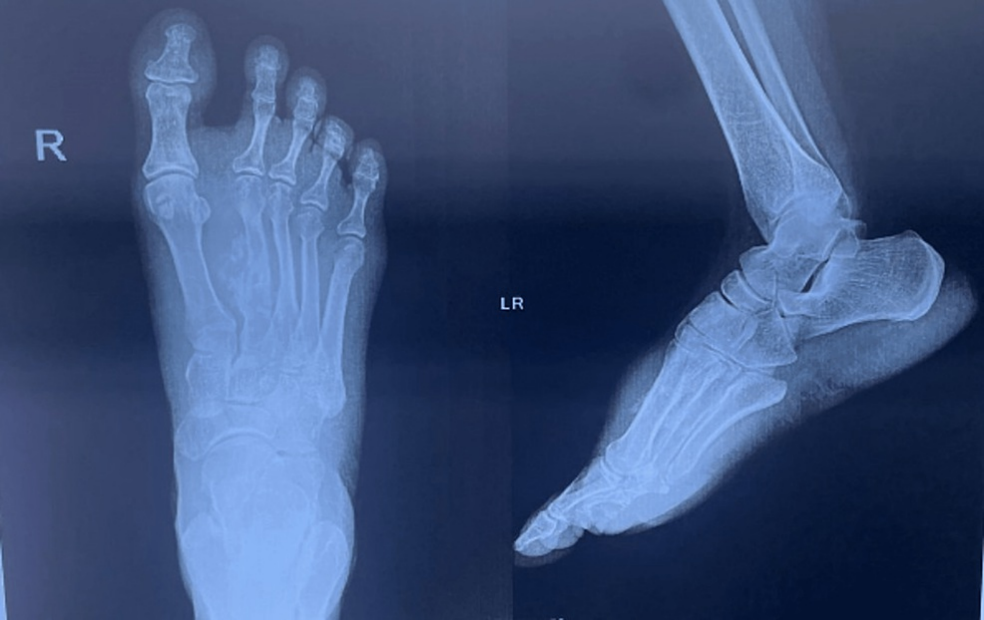
Preoperative X-ray showing an expansive cortical diaphyseal lesion in the right second metatarsal with punctate calcification and periosteal reaction

A 47-year-old woman presented to the surgical oncology outpatient department with a painful, progressive swelling on the dorsum of the right foot of two months duration. She had no history of trauma or fever. The patient had a 5x3 cm bony hard mass arising from the second metatarsal bone of the right foot on the dorsal aspect. There was no evidence of popliteal or inguinal lymphadenopathy and distal motor or sensory deficits. All distal pulses were equally felt. She presented us with a fine-needle aspiration cytology (FNAC) report from the soft tissue component of the metatarsal swelling, which showed features of a giant cell tumor (GCT). The plain radiograph revealed an expansive cortical diaphyseal lesion in the right second metatarsal with punctate calcification and a periosteal reaction that was suggestive of osteosarcoma (Figure 1).

**Figure 2:**
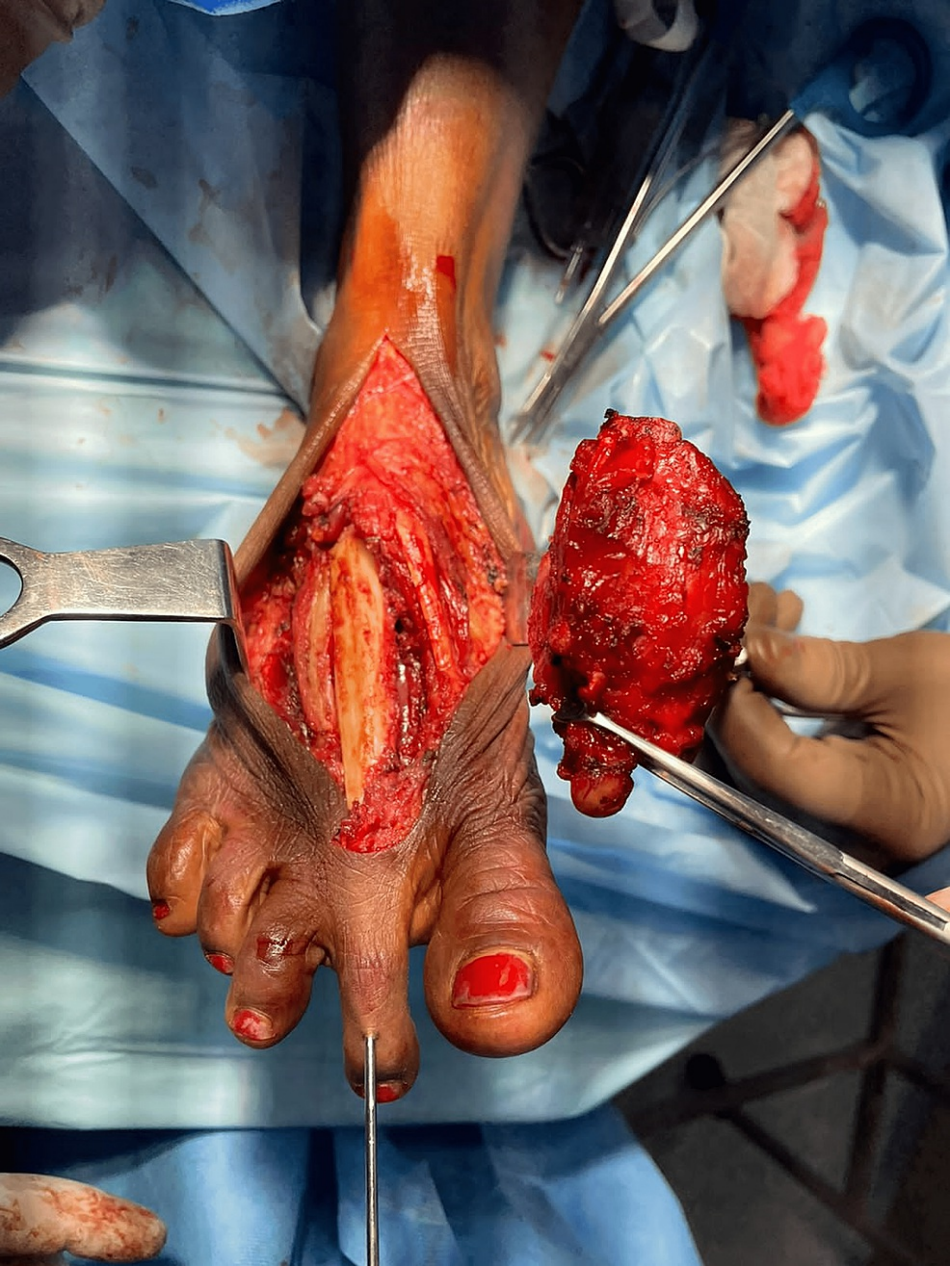
Final intraoperative image displaying the fixation of the fibular graft with a k-wire following a wide local excision (second metatarsectomy)

**Figure 3:**
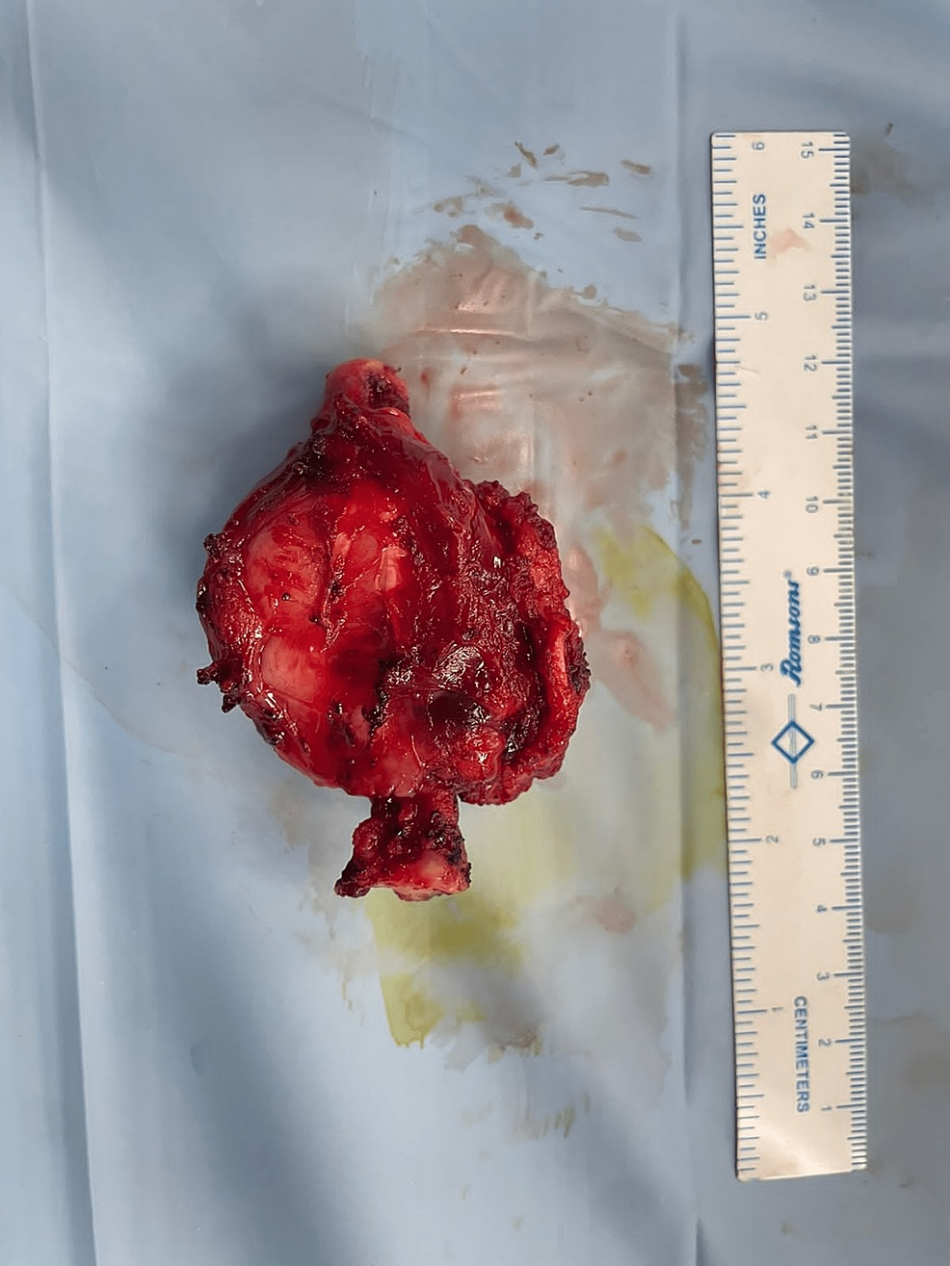
Wide local excision (metatarsectomy) specimen

The chest radiograph of this patient was normal. Due to the patient's financial constraints, an MRI of the foot could not be carried out, and we proceeded with the surgery. A wide local excision with the right second metatarsectomy was performed (R0 en-bloc resection). The foot was reconstructed using a 6 cm non-vascularized fibular graft using k-wire (Figures 2, 3).

**Figure 4:**
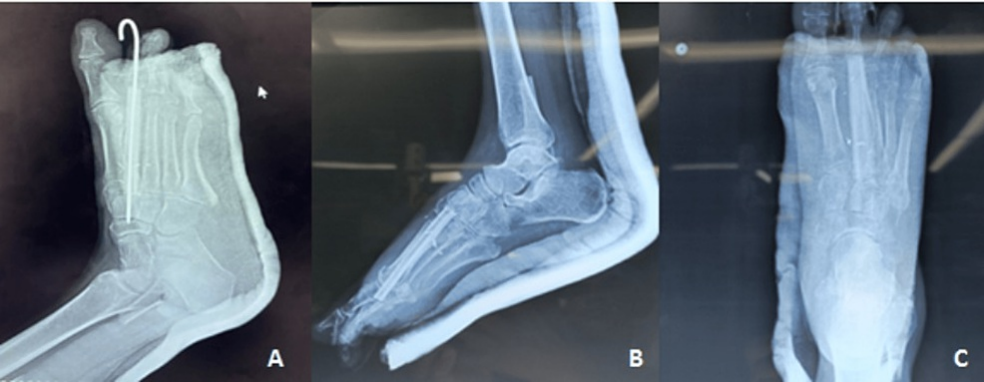
Postoperative X-ray of the foot. (A) Immediate postoperative X-ray displaying the k-wire securing the fibular implant; (B, C) X-ray taken after removal of the k-wire

The postoperative period was uneventful. On the fifth postoperative day, she was able to commence partial weight bearing, and on the 14th postoperative day, her sutures were removed. The k-wire was removed six weeks after surgery (Figure 4).

**Figure 5:**
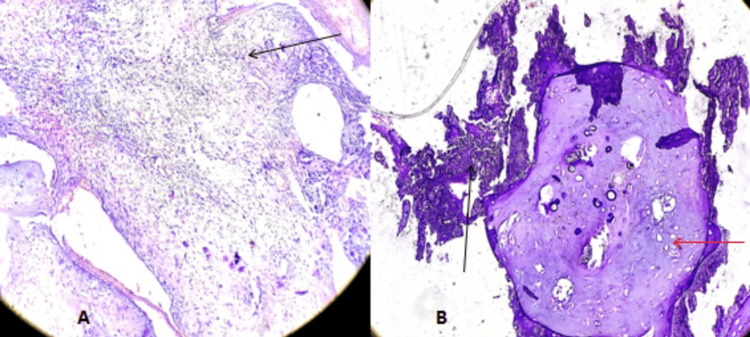
Histopathological Images. (A) sheets of spindled cells with marked pleomorphism and brisk mitosis (black arrow); (B) osteoid matrix (black arrow) as well as a chondroid matrix (red arrow)

The final histopathology report revealed a diagnosis of chondroblastic osteosarcoma with clear margins (Figure 5).

In light of the conclusive histopathology report favoring chondroblastic osteosarcoma, the patient received adjuvant chemotherapy using the MAP (high-dose methotrexate, doxorubicin, and cisplatin) protocol. As the resection margins were tumor-free, no adjuvant radiation therapy was necessary.

## Discussion

We reported the case of a 47-year-old woman with a painful swelling on the dorsum of her right foot, which was a bony, hard mass from the second metatarsal bone. FNAC suggested a GCT, although conventional radiography suggested osteosarcoma. An MRI scan was unaffordable. Wide local excision with right second metatarsectomy and non-vascularized fibular graft reconstruction were performed. Histopathology indicated chondroblastic osteosarcoma with clear margins after a smooth recovery. The patient received adjuvant chemotherapy, and no radiation therapy was required due to tumor-free resection margins.

The clinical presentation of osteosarcoma of the foot bones is non-specific. Schuster AJ et al. [4] analyzed the prediagnostic symptoms of 21 of the 23 patients with osteosarcoma of the foot and discovered that eight (38%) complained of only pain, two (10%) reported only swelling, and 11 (52%) documented both. Our patient presented primarily with painful osseous swelling. In their study of 23 patients with osteosarcoma of the foot, Schuster AJ et al. [4] analyzed the precise site of occurrence and discovered that in two patients a phalanx (9%), in five patients a metatarsal bone (21%), and in 16 patients a tarsal bone (70%) were affected. Three patients showed signs of primary metastases: two had pulmonary metastases and one had ipsilateral inguinal lymph node involvement. There were 13 men and 10 women, with a median age of 32 years (range: 6-58 years). Our case was a chondroblastic osteosarcoma of the second metatarsal bone of the right foot in a 47-year-old woman with no evidence of distant metastasis.

Radiographically, osteosarcoma can mimic benign conditions such as aneurysmal bone cysts and chondroblastomas [5]. Plain radiographs of our patient, however, revealed an expansive cortical diaphyseal lesion in the left second metatarsal with punctate calcification and periosteal reaction, which are suggestive of osteosarcomas. Classic osteosarcoma characteristics include aggressive cortical destruction, an ill-defined border, and a soft tissue mass. Typical radiographic findings of periosteal osteosarcoma include a broad-based soft-tissue mass affixed to the cortex (100%), cortical hypertrophy (82%), extrinsic scalloping of the cortex (91%), and a periosteal reaction (95%) [6].

Several differential diagnoses, including benign tumors, must be considered when diagnosing and treating such patients. GCTs and chondromyxoid fibroma are the two most prevalent benign tumors affecting the metatarsal [2]. GCTs can manifest as a bony enlargement and may resemble malignant lesions on imaging. Chondromyxoid fibroma can manifest as a painful enlargement, and imaging may reveal calcification. To differentiate these benign conditions from malignant tumors, histopathological examination plays a critical role [2]. The diagnosis of osteosarcoma in our case was initially challenging, as the patient presented with a swelling on the dorsum of the foot, which clinically mimicked a benign GCT, and FNAC from the soft tissue component of the swelling indicated features of a GCT. This emphasizes the importance of histopathological examination, as the final diagnosis revealed chondroblastic osteosarcoma. Due to the rarity of foot osteosarcoma, little is known about patient and tumor characteristics; in addition, the prognosis may be poor due to delayed presentation and misdiagnosis. A delayed diagnosis of osteosarcoma, regardless of location, may not only reduce long-term survival but also modify the treatment plan, resulting in less favorable functional and cosmetic outcomes. Initial misdiagnoses of foot osteosarcoma are common, accounting for 50% of the cases reported by Biscaglia et al. [5]. This group discovered that the average time between the beginning of symptoms and the diagnosis of osteosarcoma of the foot was greater than two years; they theorized that this was due in part to the fact that osteosarcoma in this location is typically found in elderly patients and the diagnosis is frequently missed [5]. The diagnosis of osteosarcoma of the foot is often challenging due to multiple factors. First, its rarity in this location contributes to the difficulty in recognizing and diagnosing the tumor. Furthermore, certain misleading plain film features can further complicate the accurate determination of the diagnosis [7].

Our patient underwent a wide local excision in the form of a second metatarsectomy, and reconstruction was done using a non-vascularized fibular graft. This choice was made due to its easier operative technique, shorter surgical time, and proven high union rates for patients up to 12 cm [8]. There is limited information regarding the use of non-vascularized fibula grafts in the treatment of metatarsal defects [9-11]. Utilizing non-vascularized fibular grafts after metatarsal resection is a valuable surgical technique that provides reconstructive options for patients with metatarsal bone defects. The excision of a metatarsal bone can result in a significant functional deficit and foot instability, necessitating an effective reconstruction strategy. The graft is taken from a non-weight-bearing portion of the fibula, without its blood supply preserved, and then used to replace the removed metatarsal bone. This technique offers numerous benefits, including the ability to preserve the length, alignment, and stability of the foot while maintaining its natural contour. Similar surgical procedures have been described by other investigators involving foot osteosarcoma. Alapatt JJ et al. [2] described a case of chondroblastic osteosarcoma in the second and third metatarsals in which wide excision and fibular graft reconstruction were performed. The patient's functional outcome was satisfactory, and there were no signs of recurrence. These findings demonstrate the feasibility and efficacy of wide local excision in conjunction with non-vascularized fibular graft reconstruction for the treatment of metatarsal osteosarcoma.

Based on the final histopathological diagnosis of chondroblastic osteosarcoma, adjuvant chemotherapy was recommended for our patient. In accordance with the established osteosarcoma treatment protocol, the administration of neoadjuvant and adjuvant chemotherapy has become the standard strategy for improving overall survival rates and minimizing the risk of metastasis [4]. Due to the lack of diagnostic certainty surrounding the tumor in our case, neoadjuvant chemotherapy was not initially administered. However, after obtaining the final histopathology report following definitive surgery, which provided the definitive diagnosis, it was determined to proceed with adjuvant chemotherapy. The inclusion of chemotherapy in our patient's treatment plan is consistent with the findings of previous studies [2,4], demonstrating its efficacy in the management of foot osteosarcoma. By implementing adjuvant chemotherapy, we aimed to optimize therapeutic outcomes and provide the patient with the most comprehensive and beneficial treatment approach for her chondroblastic osteosarcoma. Chondroblastic osteosarcoma is generally classified as a low- to intermediate-grade surface osteosarcoma [12] with a favorable prognosis.

It is crucial to acknowledge the limitations of our study, including the lack of MRI due to financial constraints. The MRI provides vital information about the tumor's extent and facilitates surgical planning. Despite this limitation, we were able to achieve clear surgical margins with wide local excision. Furthermore, the follow-up period in our case is relatively short, and long-term outcomes are yet to be determined.

## Conclusions

In conclusion, this case report illustrates the challenges associated with diagnosing and treating chondroblastic osteosarcoma involving the metatarsal bone. Comparisons with similar studies highlight the significance of an accurate histopathological diagnosis, wide local excision with reconstruction, and adjuvant chemotherapy in attaining favorable outcomes. To optimize treatment for metatarsal osteosarcoma, additional research and larger-scale studies need to be conducted to expand our understanding of this rare entity and optimize treatment options.
